# Advances in orthodontic treatment for periodontal disease: a bibliometric analysis, emerging insights and clinical implications

**DOI:** 10.3389/fdmed.2025.1600672

**Published:** 2025-07-24

**Authors:** Jiahui Zhao, Zezhou Feng, Yujiang Liu, Silu Sun, Zhiyuan Feng

**Affiliations:** ^1^ Department of Stomatology, Xinzhou People's Hospital, Xinzhou, China; ^2^ Shanxi Medical University School and Hospital of Stomatology, Taiyuan, China; ^3^ Department of Orthodontics, Shanxi Provincial People’s Hospital, Shanxi Medical University School and Hospital of Stomatology, Taiyuan, China

**Keywords:** orthodontic, periodontal disease, quality of life, translational medicine, clinical application

## Abstract

**Objectives:**

This study aims to perform a comprehensive bibliometric analysis of research on orthodontic treatment for patients with periodontal disease. By examining publication trends, citation patterns, and research hotspots, we seek to understand the evolution of this field, identify future trends, and promote an integrated approach to enhance treatment outcomes and patient care.

**Methods:**

A comprehensive literature search was performed using the Web of Science and PubMed databases with the keywords “periodontal disease” “periodontology” “periodontitis” and “orthodontics”.The retrieved records were systematically analyzed and visualized utilizing CiteSpace 6.2 and VOSviewer software. Bibliometric analysis was conducted across multiple dimensions, including authorship, geographic distribution (countries and institutions), keyword trends, and citation patterns in relevant journals.

**Results:**

After screening titles, abstracts, and keywords, a total of 580 articles met the inclusion criteria for further analysis. The leading publishing countries were China and USA with significant contributions from Grigore T. Popa University of Medicine & Pharmacy. Ionuț Luchian emerged as the most prolific author, while Ainamo J. demonstrated considerable influence based on citation metrics. Authoritative journals, such as the American Journal of Orthodontics and Dentofacial Orthopedics, were identified as the most frequently cited publications in this field.

**Conclusions:**

Future research in orthodontic treatment for patients with periodontal disease is expected to focus on developing personalized treatment plans, utilizing innovative biomaterials, designing advanced biomarkers and predictive models, integrating regenerative medicine approaches, and comprehensively assessing patients’ overall quality of life. These directions aim to enhance treatment efficacy, improve patient outcomes, and ensure a more holistic and individualized approach to care.

## Clinical significance

Our bibliometric findings point to five synergistic domains that can directly inform and enhance clinical management of periodontally compromised patients undergoing orthodontic therapy:

1. Precision-Guided Treatment Planning
  a. Incorporate genetic and biomarker screening (e.g., SNP panels, salivary cytokines) into initial assessments to stratify patients by risk of root resorption or periodontal breakdown.
  b. Use machine-learning–driven predictive models to tailor force levels, torque prescriptions, and treatment duration-minimizing adverse events in high-risk individuals.
2. Advanced Biomaterials and Digital Workflows
  a. Adopt 3D-printed, antimicrobial polymer brackets or shape-memory archwires in patients with active inflammation to reduce plaque retention and improve comfort.
  b. Utilize CAD/CAM–generated aligners or custom appliances designed via intraoral scanning and FEA to ensure individualized force application and better control of tooth movement trajectories.
3. Microbiome-Centered Prevention
  a. Implement routine monitoring of subgingival microbiota (e.g., 16S rRNA profiling) before and during treatment to detect dysbiosis early.
  b. Supplement mechanical debridement with site-specific probiotics or synbiotics to restore microbial balance and blunt inflammatory flares around brackets.
4. Regenerative Support Strategies
  a. In cases of existing periodontal defects or delayed movement, coordinate orthodontic activation with local application of MSC-laden scaffolds, PRF membranes, or gene-activated matrices to promote ligament and bone regeneration.
  b. Schedule adjunctive growth-factor injections (e.g., rhPDGF-BB) in the most compromised sites to accelerate tissue healing and stabilize periodontium.
5. Holistic, Patient-Centered Care
  a. Integrate validated patient-reported outcome measures (OHIP-14, PIDAQ) into routine visits to monitor comfort, anxiety, and satisfaction.
  b. Offer pre-treatment virtual-reality simulations or digital counseling apps to reduce dental anxiety, improve adherence, and personalize communication around expected outcomes.

## Introduction

Periodontal disease, encompassing conditions such as gingivitis and periodontitis, represents a significant public health concern worldwide due to its high prevalence and potential to cause tooth loss, impaired mastication, and diminished quality of life (1–3). Chronic inflammation of the supporting structures of the teeth not only affects oral health but has also been linked to systemic conditions including cardiovascular disease, diabetes, and adverse pregnancy outcomes (4–6). Effective management of periodontal disease is therefore crucial, necessitating comprehensive approaches that address both the microbial and host-response aspects of the disease (7–9).

Orthodontic treatment, traditionally focused on the alignment of teeth and correction of malocclusions, has increasingly been recognized for its role in optimizing periodontal health (10–13). Properly aligned dentition can enhance plaque control, reduce areas susceptible to periodontal breakdown, and distribute occlusal forces more evenly, thereby mitigating the progression of periodontal disease (14, 15). Conversely, periodontal status can influence orthodontic outcomes, as severe periodontal disease may limit the feasibility and timing of orthodontic interventions (16). This bidirectional relationship underscores the necessity for integrated treatment planning that considers both orthodontic and periodontal perspectives.

Moreover, in periodontal patients undergoing orthodontic therapy, the re-establishment of optimal occlusal relationships and improved dental alignment can have a significant impact on temporomandibular joint (TMJ) health. By harmonizing occlusal contacts and promoting even force distribution across the dental arches, orthodontic interventions may alleviate parafunctional loading and reduce TMJ strain—potentially mitigating symptoms such as joint pain, clicking, or limited range of motion. Conversely, inadequate control of tooth movement in a compromised periodontal foundation can exacerbate joint loading imbalances and provoke or perpetuate temporomandibular disorders. Thus, integrated treatment planning that concurrently addresses periodontal status, occlusion, and TMJ mechanics is essential to optimize both periodontal and temporomandibular outcomes.

Despite broad acknowledgment of the interplay between orthodontic and periodontal therapies, studies in this multidisciplinary field remain fragmented—exhibiting disparate emphases, methodologies, and geographic distributions. Understanding the evolution, current trends, and future directions of research at this intersection is essential for identifying knowledge gaps, fostering collaborations, and guiding evidence-based clinical practices. Bibliometric analysis serves as a powerful tool to quantitatively evaluate the existing literature, revealing patterns in publication output, influential authors and institutions, prevalent research themes, and citation networks (17–24).

Notably, unlike narrative or systematic reviews that depend on subjective selection criteria and yield primarily qualitative syntheses, a bibliometric approach provides a rigorous, data-driven map of the scientific terrain. By analyzing co-authorship and co-citation networks, tracking keyword co-occurrence and burst phenomena, and visualizing institutional and geographic collaborations, bibliometric methods expose the underlying structure and dynamic evolution of a research field. This quantitative perspective uncovers emerging hotspots, reveals interdisciplinary linkages, and highlights underexplored domains—insights that traditional literature reviews alone cannot deliver.

To date, limited bibliometric studies have comprehensively mapped the research on orthodontic treatment in patients with periodontal disease, thereby impeding the ability to synthesize knowledge and identify emerging areas of interest. This study aims to fill this gap by conducting a bibliometric analysis of publications indexed in the Web of Science and PubMed databases. Utilizing advanced visualization tools such as CiteSpace and VOSviewer, we analyze trends in authorship, geographic distribution, keyword prevalence, and citation patterns. By elucidating the intellectual structure and dynamic developments within this field, this research seeks to inform future investigations, enhance interdisciplinary collaboration, and ultimately improve clinical outcomes for patients requiring combined orthodontic and periodontal care.

## Materials and methods

### Database

Web of Science (WOS) and PubMed are crucial databases in bibliometric analysis, each offering unique strengths. WOS is renowned for its comprehensive coverage across hard sciences, social sciences, and arts, providing robust citation data and high-quality, peer-reviewed sources (23). PubMed excels in the biomedical field, offering free access to a vast repository of life sciences and medical literature, ensuring up-to-date and reliable information. Together, these databases provide a well-rounded, multidisciplinary foundation for thorough and reliable bibliometric studies.

### Search strategy

A systematic literature search was conducted on February 10, 2025, utilizing the Web of Science and PubMed databases. The search strategy employed a combination of key terms including “periodontal disease,” “periodontology,” “periodontitis,” and “orthodontics” to identify relevant original research and review articles. No publication date restrictions were imposed to ensure comprehensive inclusion of all eligible documents available through the search date. It is important to note that publications from 2025 reflect partial-year coverage (January to February) and therefore were excluded from longitudinal trend analyses to avoid overinterpretation of annual research patterns.

### Data screening and collection

To ensure the quality and reliability of the literature, we systematically searched the Web of Science (WOS) and PubMed databases, applying filters to exclude non-peer-reviewed publications. Three independent researchers conducted a rigorous screening process: initially evaluating titles, keywords, and abstracts, followed by a thorough full-text review to identify the most relevant studies for inclusion. All selected articles, including complete bibliographic information and reference lists, were downloaded in plain text format for subsequent analysis. For comprehensive bibliometric analysis, we employed two specialized software tools: Citespace and VOSviewer. These enabled us to perform detailed network analyses, including co-occurrence and co-citation mapping. Our analysis encompassed annual publication trends, contributing countries and institutions, author contributions, keywords, and journals within the field.

### Limitations

Several methodological constraints may affect the breadth and balance of our analysis. First, we restricted retrieval to the Web of Science Core Collection and PubMed, which—while comprehensive—do not cover all relevant outlets. Key articles indexed exclusively in Scopus, Embase, CNKI, Dentistry & Oral Sciences Source, or other specialty repositories may have been omitted, potentially under-representing certain regions or disciplines. Second, by limiting our search to English-language records, we likely excluded high-quality studies published in other languages (e.g., Chinese, Spanish, Portuguese), introducing language bias and skewing geographic and institutional trends. Third, bibliometric outcomes hinge on search-term selection and Boolean operators; although our query was iteratively refined to include major MeSH terms and synonyms for “periodontal disease” and “orthodontics,” alternative descriptors or emerging keywords may fall outside our scope. These limitations could lead to underestimation of publication counts in specific subfields and regions. Future updates should broaden database coverage, incorporate non-English search terms, and include gray-literature sources to achieve a more inclusive, representative mapping of the orthodontic–periodontal research landscape.

### Statistical analysis

The core of this study focused on the utilization of numerical values and corresponding percentages to depict the statistical indicators. Importantly, no comparative analyses were conducted, thus eliminating the need for establishing a significance level.

## Results

### General information

**Figure 1:**
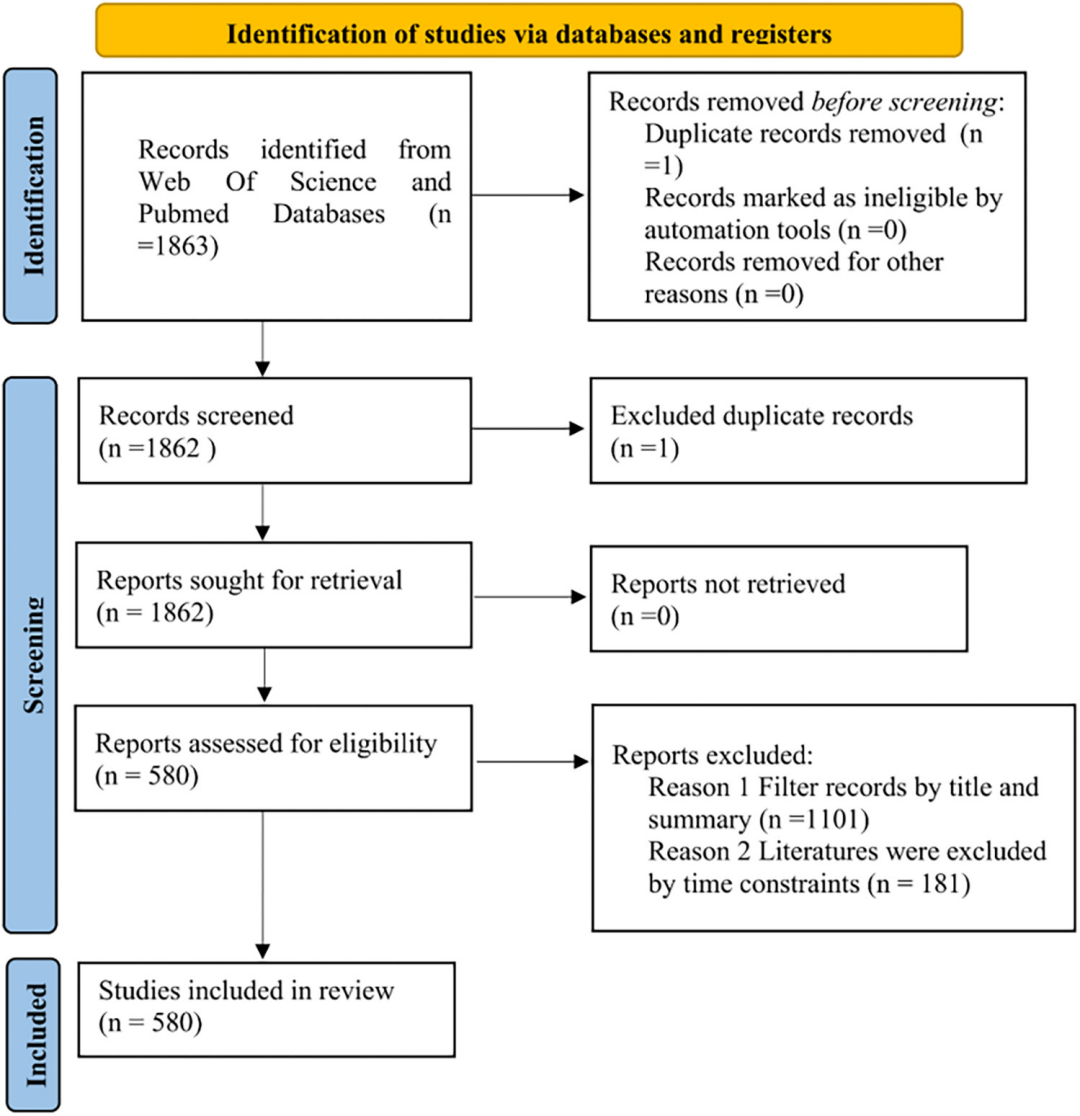
Data screening.

**Figure 2:**
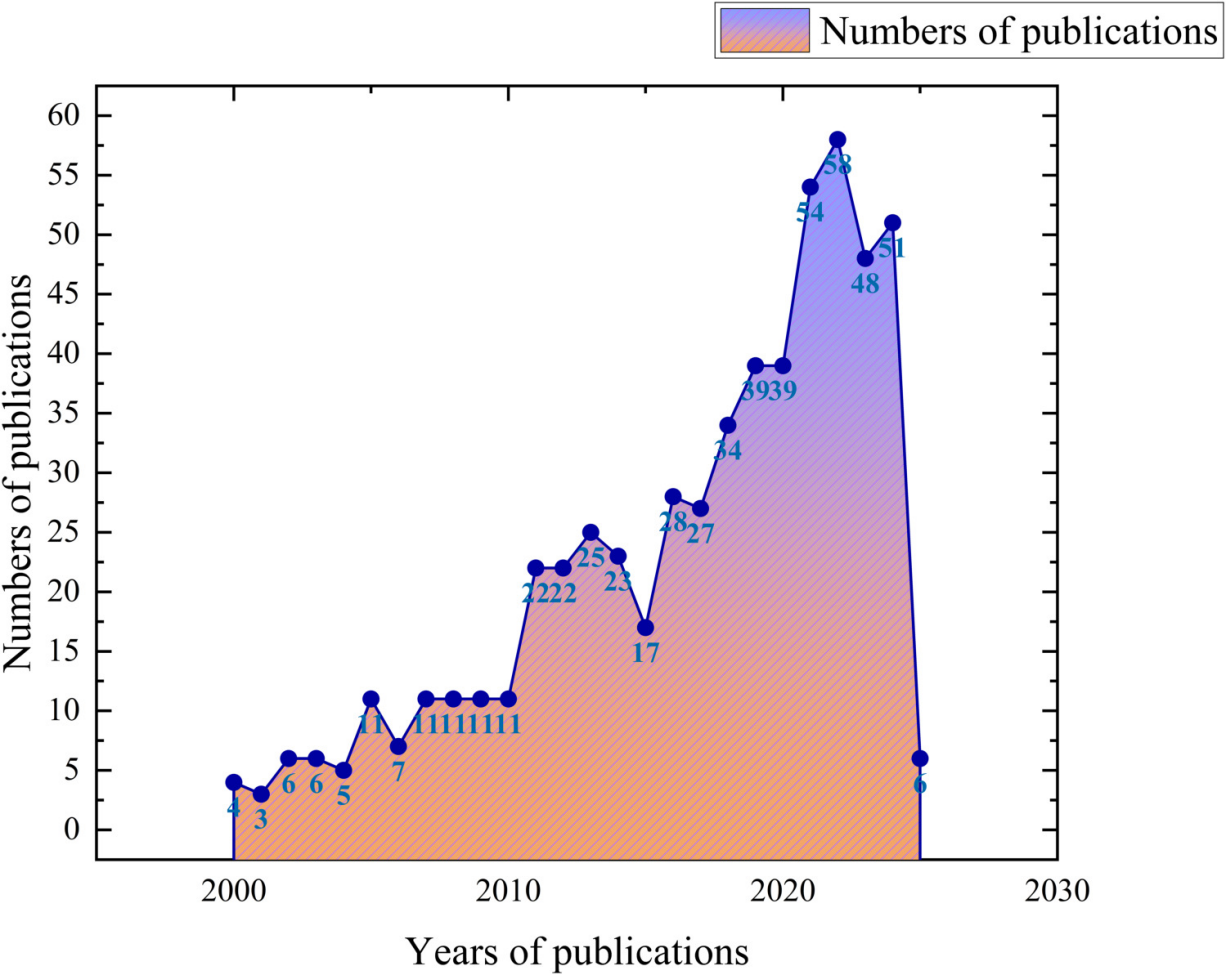
Annual changes in the number of articles published. Annotation: 1. In bibliometric analysis, a node with a high school centrality score means that it plays an important role in connection and communication in the research field, which may be the core research or the research with greater influence in the field. 2. In the various types of co-occurrence plots presented, the color of the nodes has no impact on the analysis outcomes. The size of the nodes, however, serves as a visual indicator of the prominence or engagement with specific literature or research topics over designated time periods. Each node represents a particular time frame, with its size generally correlating to the number of citations or identified relationships during that span. Consequently, larger nodes signify that a node—be it a country, institution, author, keyword, or article—has received substantial citation or discussion, reflecting its influence and significance within the academic community.

In total, 762 articles were included in our analysis, comprising 695 original research papers and 67 review articles. Given that the software analysis was configured for the period from 2000 to 2025, we ultimately focused on 580 articles for detailed examination (refer to Figure 1). The annual publication rate in this domain was relatively low prior to 2010. However, beginning in 2010, a linear regression analysis of publication counts (2010–2022) revealed a statistically significant upward trend, indicating a steady increase that culminated in a peak in 2022 (refer to Figure 2).

### Country analysis

**Figure 3:**
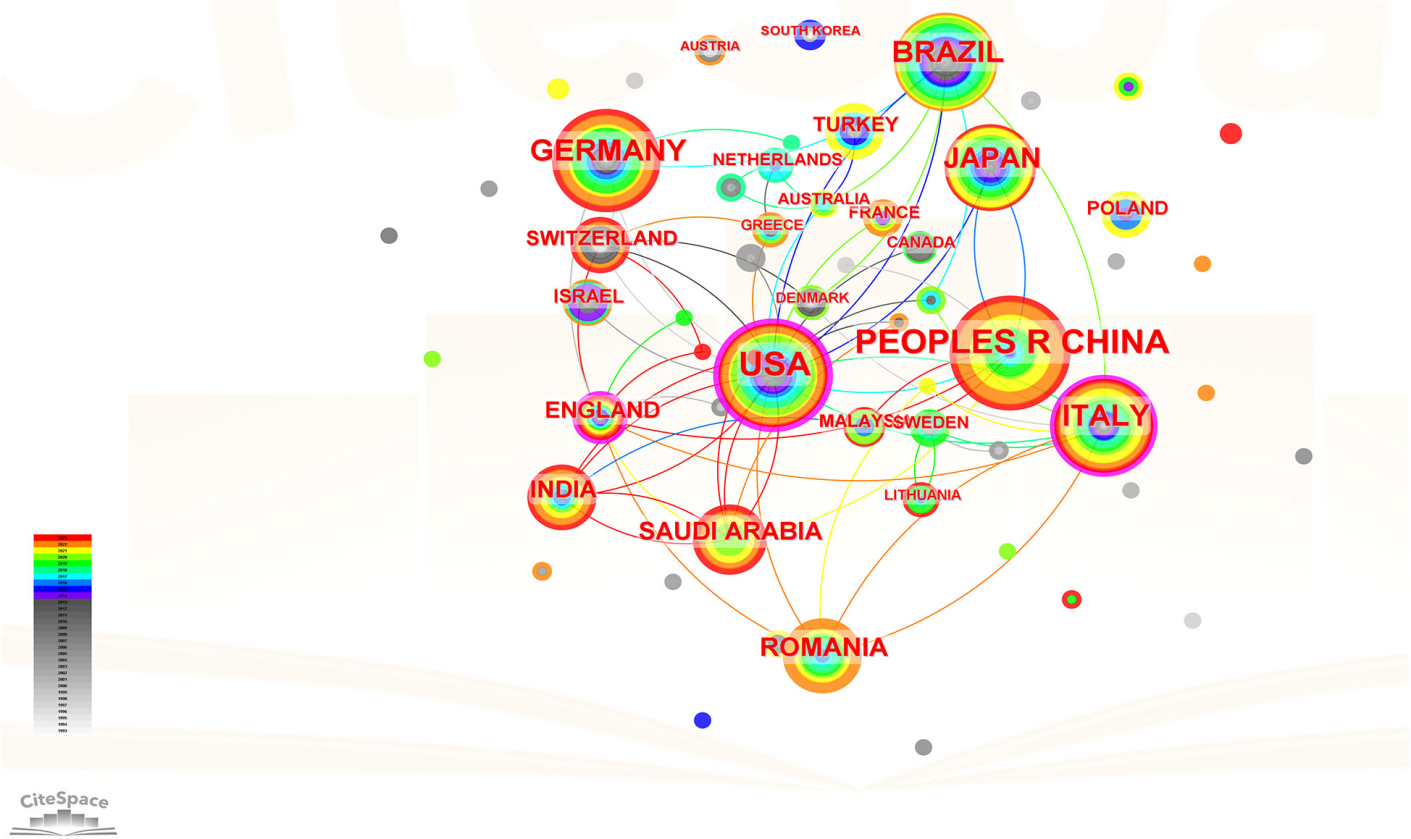
Co-occurrence map of countries.

We used Citespace 6.2 software to analyze the countries of the target literature. The visualization graph displays 61 nodes and 69 links (see Figure 3), indicating that the relevant literature originates from 61 countries, with 69 instances of co-authorship between any two countries. Statistical analysis identified China, the USA, Germany, Brazil, and Italy as the top five countries in terms of publication output (see Table 1). The centrality score highlights countries' ability to collaborate internationally and their influence in the field. The USA, Italy, England, Australia, and Switzerland ranked highest in centrality scores, with the USA leading, demonstrating its significant collaborative capacity (see Table 2).

**Table 1:** The top five countries in terms of the number of published papers on periodontal and orthodontic research.

Rank	Countries	Frequency
1	China	68
2	USA	67
3	Germany	44
4	Brazil	42
5	Italy	37

**Table 2:** The top five countries with the highest academic influence and attention in the field.

Rank	Countries	Centrality
1	USA	0.36
2	Italy	0.15
3	England	0.12
4	Australia	0.08
5	Switzerland	0.08

Annotation: Centrality quantifies how often a country lies on the shortest paths connecting pairs of other countries:• A high centrality score indicates that the country serves as a bridge or broker, linking otherwise disconnected regions of the network and facilitating information flow. • Countries with elevated centrality often play an outsized role in shaping research agendas, mediating international collaborations, and disseminating innovations across scientific communities. • By contrast, countries with low centrality may produce substantial output (high publication counts) but participate primarily in more localized or insular collaboration clusters.

### Institution analysis

**Figure 4:**
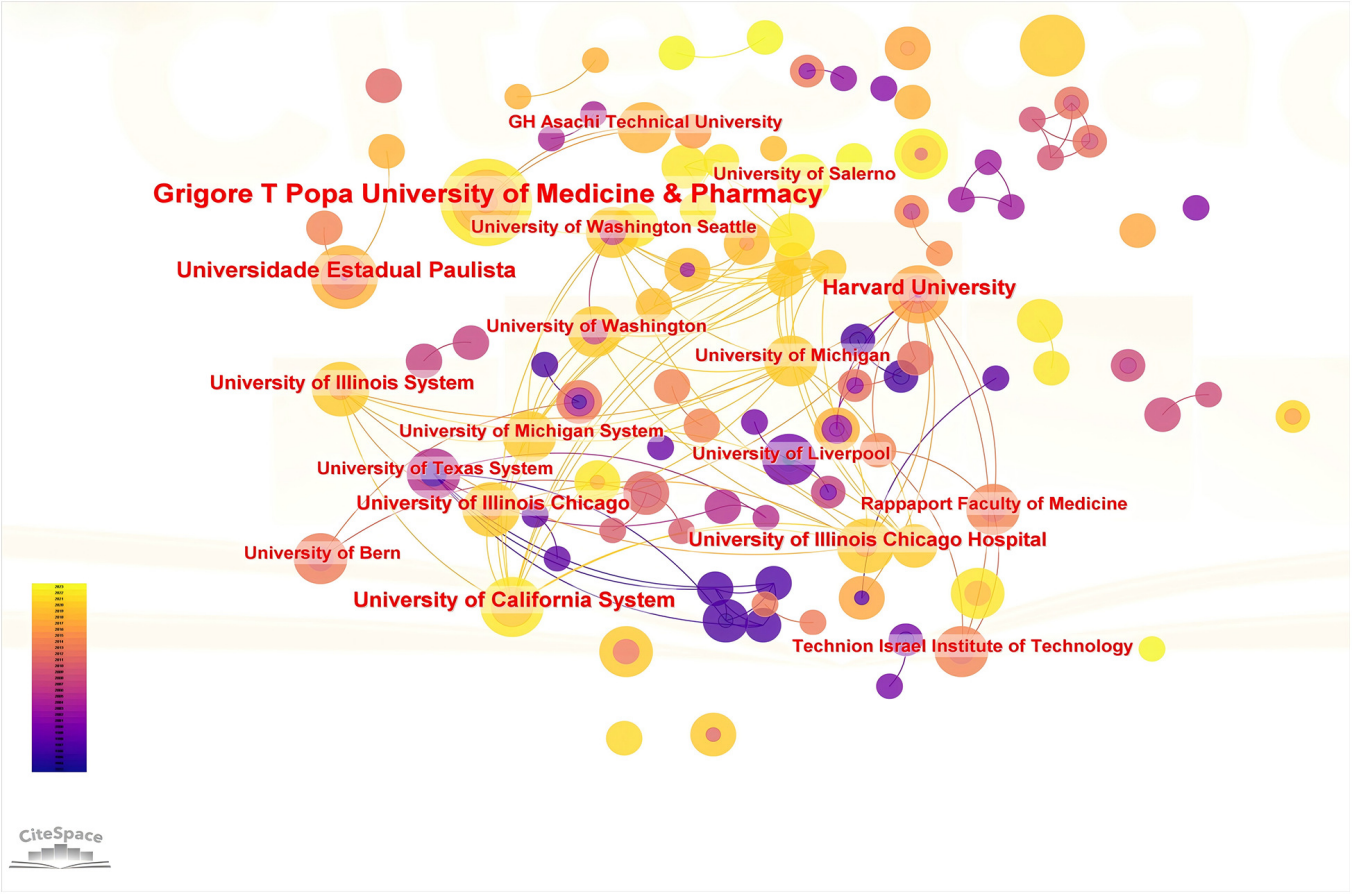
Co-occurrence map of research institutions.

CiteSpace 6.2 software was used to analyse institution, the visualization graph consists of 160 nodes, with 122 connecting lines between them (see Figure 4). Notably, the target literature in our study was sourced from a diverse range of 160 institutions, and we observed instances where any two institutions appeared together in the same literature for a total of 122 occurrences. The top 5 institutions in terms of the number of publications were Grigore T Popa University of Medicine & Pharmacy, Universidade Estadual Paulista, Harvard University, King Khalid University and University of California System (see Table 3). In the institutional centrality score, Harvard University has the highest centrality score, which demonstrates its academic focus and influence in the field. (see Table 4).

**Table 3:** The top five institutions in terms of the number of published papers in this field.

Rank	Institutions	Frequency
1	Grigore T Popa University of Medicine & Pharmacy	12
2	Universidade Estadual Paulista	7
3	Harvard University	6
4	King Khalid University	6
5	University of California System	6

**Table 4:** The top five most influential institutions in this research field.

Rank	Institutions	Centrality
1	Harvard University	0.20
2	Grigore T Popa University of Medicine & Pharmacy	0.17
3	Universidade Estadual Paulista	0.13
4	King Khalid University	0.10
5	University of California System	0.06

Frequency: Number of published papers in the field.

Centrality: The academic attention and influence of the institution.

**Table 5:** Leading contributors in orthodontic-periodontal research.

Rank	Authors	Frequency
1	Luchian Ionut	6
2	Martu Silvia	6
3	Martu Ioana	6
4	Mandelaris George A	6
5	Vata Ioana	5

**Table 6:** The top five highly cited researchers in orthodontic-periodontal studies.

Rank	Authors	Frequency
1	[ANONYMOUS]	73
2	WENNSTROM JL	60
3	MELSEN B	53
4	ARTUN J	41
5	BOLLEN AM	38

**Table 7:** The most academically influential cited author in the field (centrality: the author's influence in the academic field).

Rank	Authors	Centrality
1	AINAMO J	0.26
2	LOE H	0.16
3	MELSEN B	0.16
4	ARTUN J	0.15
5	BOLLEN AM	0.15

### Author and cited author analysis

**Figure 5:**
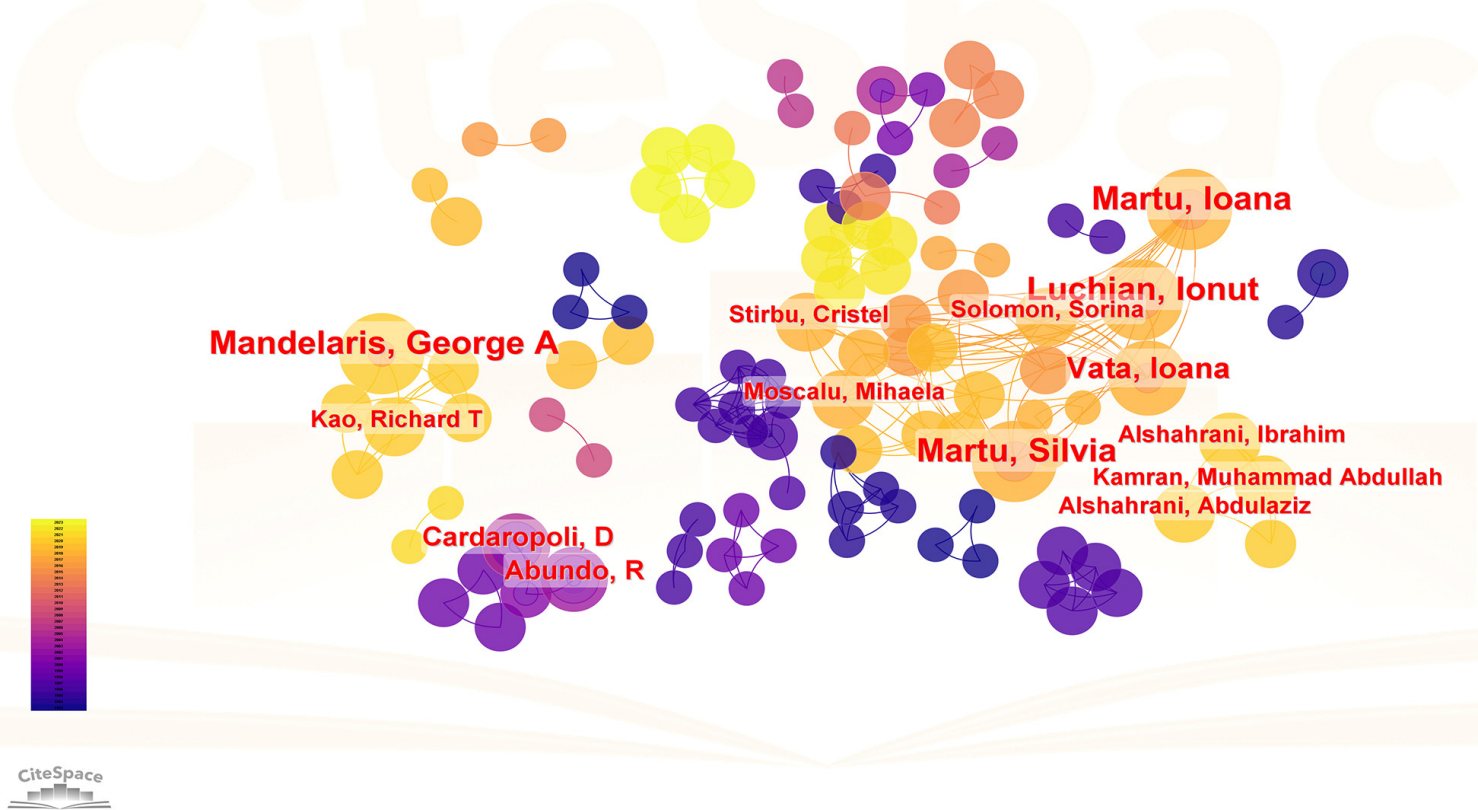
Author co-authored visualization map.

**Figure 6:**
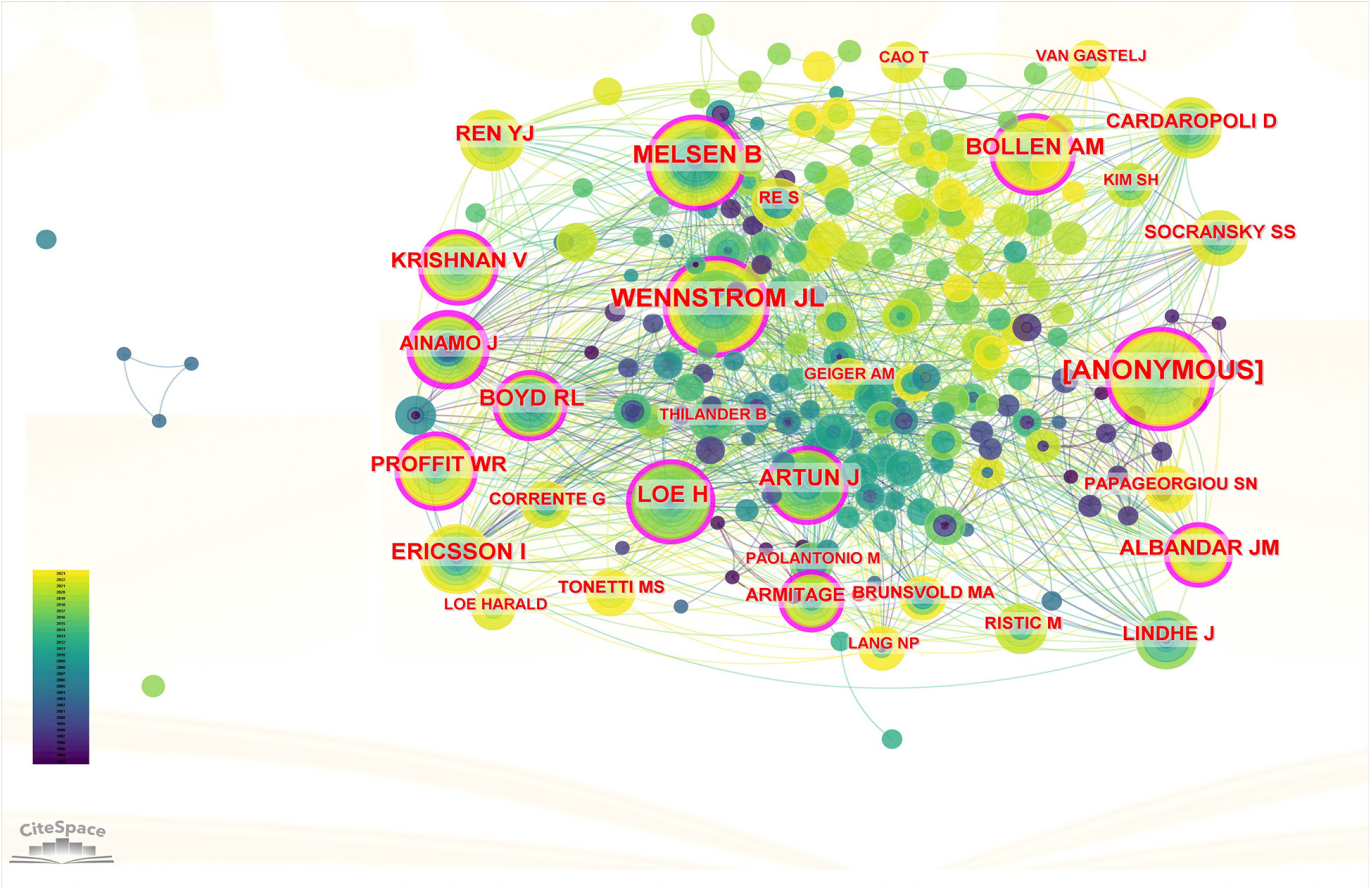
Author co-citation visualization map.

In CiteSpace, author analysis identifies key contributors, collaboration networks, and research trends within a field, highlighting influential researchers. Cited author analysis, on the other hand, evaluates the impact and recognition of individuals by examining citation frequencies, unveiling foundational works and thought leaders who shape the discipline. Both analyses provide insights into academic influence and collaborative dynamics. The visualization graph consists of 160 nodes, with 122 connecting lines between them. Notably, the target literature in our study was sourced from a diverse range of 160 authors, and we observed instances where any two authors appeared together in the same literature for a total of 122 occurrences (see Figure 5). The top 5 authors in terms of the number of published papers were Luchian Ionut, Martu Silvia, Martu Ioana, Mandelaris George A and Vata Ioana (see Table 5). As depicted in Figure 5, these authors primarily conducted independent research within their respective institutions or countries. Next, we performed author co-citation analysis. Notably, the top 5 co-cited authors comprised [ANONYMOUS], WENNSTROM JL, MELSEN B, ARTUN J and BOLLEN AM (see Table 6). Among the cited authors, the top five authors with centrality scores were AINAMO J, LOE H, MELSEN B, ARTUN J, and BOLLEN AM (see Table 7). AINAMO J has the highest score, which indicates that he has not only extensive cooperation but also great influence in this field (see Figure 6).

### Keyword analysis

In CiteSpace, keyword analysis serves crucial functions by identifying research hotspots and emerging trends within a field. By evaluating keyword occurrences and co-occurrences, researchers can detect central themes and influential studies, shedding light on the intellectual structure and dynamic evolution of the literature. Keyword analysis also helps uncover interdisciplinary links and potential collaboration opportunities across different research domains. Moreover, it informs future research directions by highlighting underexplored areas and guiding researchers toward novel and impactful investigations.

### Research hotspot and future trend analysis

**Figure 7:**
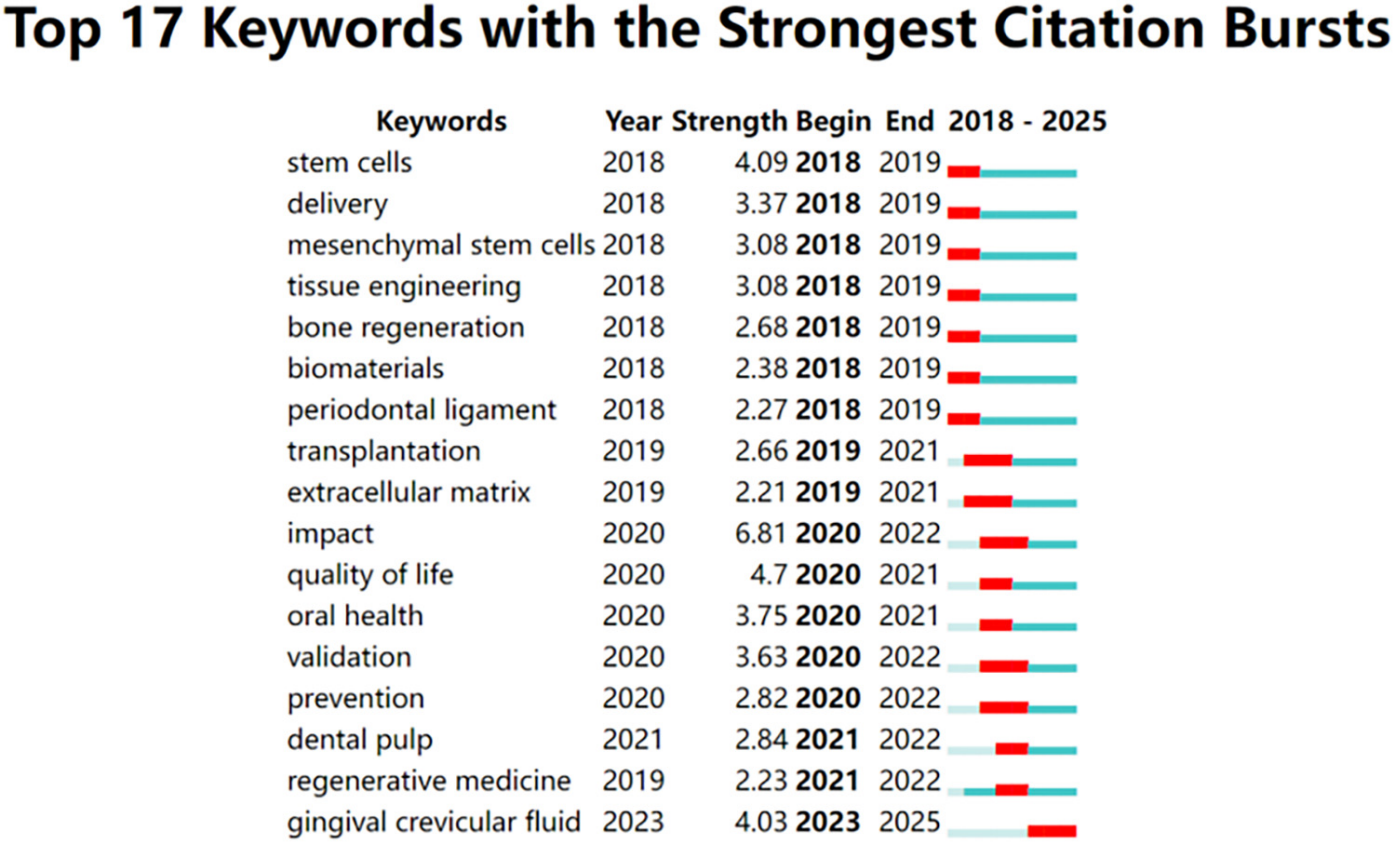
Burst graph of keywords. Note to Figure: Among the multitude of keywords examined, a notable selection of 11 emerged distinguished by their significant citation bursts. These keywords exhibited pronounced peaks denoted by red lines, symbolizing the years when they were prominently employed. Conversely, green lines signify periods within the timeframe from 1993 to 2023 when these keywords were less frequently utilized.

According to the analysis (see Figure 7), the current research hotspots in this field focus on the following directions:

1. Personalized treatment plan: Develop a personalized orthodontic treatment plan based on the patient's genetic background, periodontal status, and biomarkers to improve treatment outcomes and reduce complications.
2. Biomaterials and technological innovation: Explore new biocompatible materials and advanced orthodontic technologies, such as 3D printing and digital orthodontics, to improve the accuracy and comfort of treatment.
3. Microbiome and Immune response research: In-depth study of oral microbiota changes and their impact on immune response in periodontal disease patients during orthodontic treatment to develop more effective prevention strategies.
4. Biomarkers and predictive models: The use of biomarkers and machine learning technology to establish predictive models to assess the risk and prognosis of orthodontic treatment for patients with periodontal disease, and improve the scientific nature of clinical decision-making.
5. Regenerative Medicine and Tissue engineering: Research periodontal tissue regeneration techniques, such as stem cell therapy and growth factor application, to promote periodontal tissue repair and improve the success rate of orthodontic treatment.
6. Patients’ quality of life and psychological factors: To evaluate the impact of orthodontic treatment on periodontal disease patients’ quality of life and psychological state, and develop a more humane treatment plan.

### Advances in orthodontic treatment identified through keyword analysis

Building on our keyword-driven analysis, recent innovations are converging to offer safer, more effective orthodontic care for patients with existing periodontal disease:

1. Precision Force Control via Smart Appliances
  • Sensor-Integrated Brackets and Aligners: Miniaturized force sensors embedded in brackets or clear aligners feed real-time data into AI-driven controllers, enabling micro-adjustments of orthodontic forces to stay within safe thresholds for compromised bone and ligament tissue.
  • Closed-Loop Biomechanical Simulation: Digital-twin models of each patient's dento-periodontal complex, built from CBCT scans and periodontal probing data, allow virtual trialing of force vectors before clinical activation—minimizing iatrogenic attachment loss.
2. 3D-Printed, Biofunctionalized Biomaterials
  • Antimicrobial & Anti-Inflammatory Surface Coatings: Customized, 3D-printed brackets and auxiliaries are now being coated with slow-release agents (e.g., silver nanoparticles, chlorhexidine) or pro-resolving lipid mediators to suppress biofilm overgrowth and local cytokine surges.
  • Bioresorbable Polymer Appliances: Shape-memory polymers that gradually degrade over the course of treatment reduce the need for appliance removal and decrease plaque-retentive hardware in the gingival sulcus.
3. Integrated Regenerative Therapies
  • Gene-Activated Scaffolds: Orthodontic anchorage devices and membrane systems are being loaded with osteogenic microRNAs or BMP-2 plasmids to promote alveolar bone remodeling *in situ*, enhancing both tooth movement and periodontal regeneration.
  • Stem Cell–Enriched Gels: Injectable, photo-crosslinkable nanogels carrying mesenchymal stem cells (MSCs) or platelet-rich fibrin have been applied alongside tooth-movement sites to accelerate ligament repair and reduce post-force inflammation.
4. Microbiome-Targeted Adjuncts
  • Probiotic/Synbiotic Delivery Systems: Aligners and power chains are being engineered to release tailored probiotic strains directly into the sulcus, stabilizing beneficial microbial communities and preventing dysbiotic shifts during orthodontic therapy.
  • Organoid and “Gum-on-a-Chip” Screening: Microfluidic periodontal models guide rapid testing of novel anti-inflammatory and antimicrobial agents under simulated orthodontic force, speeding translation of new adjuncts into clinical trials.
5. Digital Monitoring & Patient Engagement
• Remote Periodontal Health Dashboards: Smartphone apps linked to intraoral cameras and wearable gingival sensors allow continuous monitoring of probing depths, bleeding on probing, and patient-reported discomfort—triggering alerts for both clinician and patient if inflammatory markers rise.
• Virtual-Reality Pre-Treatment Counseling: VR simulations of anticipated tooth movement and gingival response reduce patient anxiety and improve adherence to oral-hygiene protocols throughout treatment.

### Cited journal analysis

**Figure 8:**
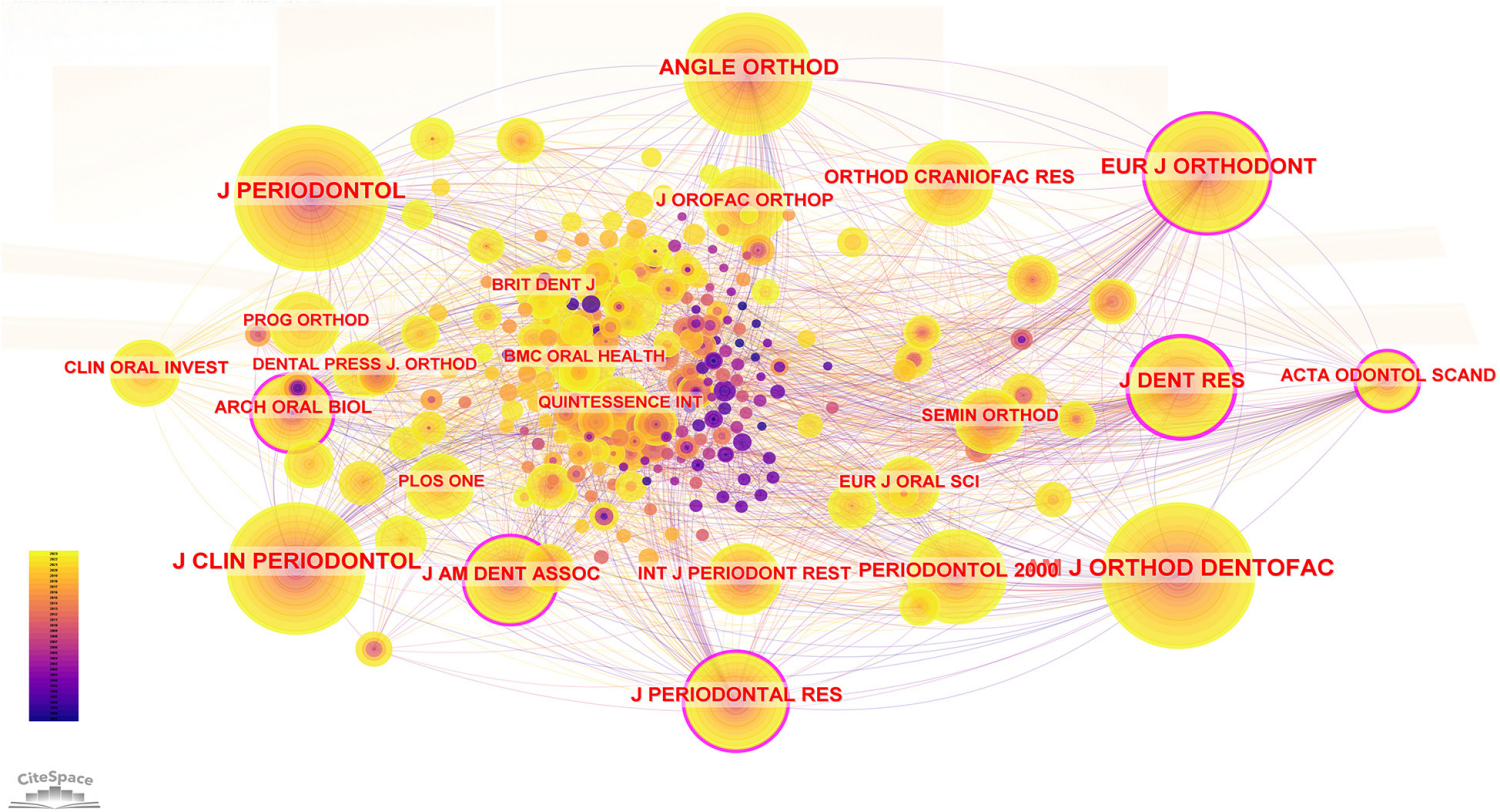
Visualization of cited journals.

The co-citation of journals reflects the correlation between various journals and disciplines. We can gain many benefits from cited journal. On the one hand, through the analysis of cited journals, we can learn about the authoritative journals in our research field to obtain more professional and credible information. On the other hand, this can help us to find the direction of submission and help us to find the most suitable journals for our research direction. In the cited journal visualization analysis plot, we can see that there are 259 nodes and 1,812 lines connecting any two nodes. This indicates that our selected literature cited 259 different journals, and the number of times any two cited journals appeared in the same literature reached 1,812 times (see Figure 8). The top five most frequently cited journals were AM J ORTHOD DENTOFAC, J PERIODONTOL, J CLIN PERIODONTOL, ANGLE ORTHOD and EUR J ORTHODONT (see Table 8). These journals are among the most authoritative in the field of orthodontics and periodontics. The top five journals with the highest centrality scores were J DENT RES, J PERIODONTAL RES, EUR J ORTHODONT, ACTA ODONTOL SCAND and ARCH ORAL BIOL (see Table 8). The centrality score of cited journals can help us to choose the most needed professional information and the best direction for submission.

**Table 8:** Authoritative journal information data table.

Rank	Cited journals	Frequency	Rank	Cited journals	Centrality
1	AM J ORTHOD DENTOFAC	415	1	J DENT RES	0.31
2	J PERIODONTOL	371	2	J PERIODONTAL RES	0.18
3	J CLIN PERIODONTOL	332	3	EUR J ORTHODONT	0.14
4	ANGLE ORTHOD	293	4	ACTA ODONTOL SCAND	0.14
5	EUR J ORTHODONT	267	5	ARCH ORAL BIOL	0.14
6	J DENT RES	230	6	J AM DENT ASSOC	0.11
7	J PERIODONTAL RES	182	7	AM J ORTHOD DENTOFAC	0.10
8	PERIODONTOL 2000	168	8	J PERIODONTOL	0.09
9	J AM DENT ASSOC	147	9	ANGLE ORTHOD	0.09
10	ORTHOD CRANIOFAC RES	123	10	SEMIN ORTHOD	0.08

## Discussion

This bibliometric analysis provides a comprehensive overview of the research landscape surrounding orthodontic treatment for patients with periodontal disease. By examining publication trends, influential authors and institutions, and prevailing research themes, the study elucidates the evolution and current state of this multidisciplinary field, while also highlighting future directions for research and clinical practice (25).

### Key findings and interpretation

Our analysis revealed a notable increase in publications on orthodontic treatment in the context of periodontal disease starting around 2010, with a peak in 2022. This surge likely reflects the growing recognition of the intricate relationship between orthodontic treatment and periodontal health, as well as advancements in technologies and methodologies that facilitate integrated treatment approaches. China, the USA, and Germany emerged as the leading contributors, underscoring the global interest and investment in this area. Institutions such as Grigore T. Popa University of Medicine & Pharmacy and Harvard University demonstrated significant output and influence, indicating their pivotal roles in advancing research and clinical practices.

The identification of key authors, notably Ionuț Luchian and Ainamo J., highlights the contributions of individual researchers in shaping the field. Ainamo J.'s high centrality score suggests a foundational influence, potentially through seminal works that have guided subsequent research and clinical protocols.

### Research hotspots and emerging trends

The keyword analysis pinpointed several critical areas of focus that reflect both current challenges and innovative solutions in the field:

1. Personalized Treatment Planning & Predictive Modeling: Emphasis on tailoring orthodontic interventions based on genetic, periodontal, and biomarker profiles signifies a shift towards precision medicine. This approach aims to enhance treatment efficacy and minimize complications by accounting for individual patient variations (26–29) The integration of biomarkers and machine learning to create predictive models represents a significant advancement in assessing treatment risks and prognoses. These tools enhance clinical decision-making by providing evidence-based predictions tailored to individual patient profiles (30–34).
  • Rationale: Standardized orthodontic protocols often fail to account for inter-individual variability in bone metabolism, inflammatory profile, and genetic susceptibility.
  • Advances: Over the past three years, keywords such as “genetic polymorphism,” “salivary biomarkers,” and “machine learning” have shown strong citation bursts. Researchers have combined single-nucleotide polymorphism (SNP) panels with serum cytokine levels to build risk-scoring models for root resorption and periodontal breakdown (35–37).
  • Emerging subthemes:

Integration of multi-omics data (genomics, proteomics, metabolomics) in predictive algorithms.

Real-time adjustment of force application based on patient-specific biomechanical simulations.

2. Biomaterials Innovation & Digital Orthodontics: The exploration of new biocompatible materials and advanced technologies such as 3D printing and digital orthodontics highlights the ongoing efforts to improve the precision, comfort, and outcomes of orthodontic treatments. These innovations not only enhance clinical practice but also contribute to patient satisfaction and compliance (38–47).
  • Rationale: Patient comfort, treatment precision, and cross-infection control drive the search for next-generation appliances.
  • Advances: The clusters “3D-printing” and “CAD/CAM” have expanded sharply since 2019. Biodegradable polymer brackets, shape-memory alloys, and antimicrobial surface coatings are under intensive investigation (48, 49). In parallel, intraoral scanners and finite-element analysis (FEA) software enable clinicians to visualize tooth-movement trajectories before applying forces (50).
  • Emerging subthemes:

Smart brackets with embedded sensors for real-time force measurement.

AI-powered treatment-simulation platforms that predict both aesthetic outcome and periodontal health impact.

3. Oral Microbiome, Immune Response & Prevention Strategies: Understanding the dynamics of oral microbiota and their interactions with the immune system during orthodontic treatment is crucial for developing effective prevention and management strategies for periodontal complications. This research underscores the importance of maintaining a healthy oral environment to support both periodontal and orthodontic health (51–60).
  • Rationale: Orthodontic appliances can disrupt biofilm ecology, altering local immune signaling and predisposing to periodontal breakdown.
  • Advances: Keywords “oral microbiota,” “inflammation markers,” and “cytokine profile” co-occur in a distinct cluster that peaked in 2021–2022. Longitudinal 16S rRNA and metagenomic studies have mapped shifts in subgingival communities during fixed-appliance therapy, linking certain pathogen surges (e.g., Porphyromonas gingivalis) to IL-1β elevation and alveolar bone loss (61, 62).
  • Emerging subthemes:

Probiotic or synbiotic adjuncts to modulate dysbiosis.

Microfluidic “gum-on-a-chip” models to screen anti-inflammatory biomaterials.

4. Regenerative Medicine & Tissue Engineering: Investigations into stem cell therapy and growth factor applications for periodontal tissue regeneration reflect the potential to not only halt disease progression but also restore damaged periodontal structures. These regenerative approaches could significantly improve the long-term success rates of orthodontic treatments in periodontally compromised patients (63–67).
  • Rationale: Iatrogenic periodontal defects and delayed tooth movement can both benefit from targeted tissue-engineering approaches.
  • Advances: The “stem cells,” “growth factors,” and “3D scaffold” clusters highlight a surge in preclinical studies using mesenchymal stem cells (MSCs), platelet-rich fibrin (PRF), and bioactive glass composites to accelerate ligament regeneration (68, 69).
  • Emerging subthemes:

Gene-activated matrices that locally deliver osteogenic miRNAs.

Bioprinting of periodontal ligament–cementum complex for *in vivo* transplantation.

5. Patient-Centered Outcomes & Psychosocial Dimensions: Assessing the impact of orthodontic treatment on patients’ quality of life and psychological well-being emphasizes a holistic approach to care. Understanding these factors is essential for developing humane treatment plans that address both physical and emotional aspects of patient health (26, 70–75).
  • Rationale: Clinician-reported measures (e.g., probing depth, tooth movement distance) overlook the patient's perceived comfort, aesthetics, and quality of life.
  • Advances: Since 2020, “oral-health-related quality of life,” “anxiety,” and “treatment satisfaction” have formed a cohesive cluster. Mixed-methods trials are now routinely incorporating validated PROM instruments (OHIP-14, PIDAQ) and semi-structured interviews to gauge adherence drivers and psychological stress (76, 77).

While all identified domains represent substantive research frontiers, their translational timelines and potential impacts warrant differentiated consideration. Based on current technological readiness and clinical demand, two areas emerge as particularly actionable:Biomaterials Innovation & Digital Orthodontics and Personalized Treatment Planning. The oral microbiome modulation and regenerative approaches, though scientifically transformative, currently face longer translational pathways due to regulatory complexities in live-biologic applications. Nevertheless, probiotic adjuvants and gene-activated scaffolds merit prioritized funding as mid-term (5–7 year) solutions for refractory periodontitis cases. Patient-centered outcome tools, while methodologically mature, require paradigm shifts in clinical evaluation standards to achieve systemic impact.

• Emerging subthemes:

Virtual-reality tools for pre-treatment counseling to reduce dental anxiety.

Longitudinal assessment of digital-app–based support networks on treatment compliance.

### Future directions

Looking forward, these five fronts are converging into a holistic, precision-digital-bio-behavioral paradigm. We anticipate:

• Closed-loop systems that marry intraoral sensor feedback with adaptive force modulation.
• Integrated dashboards combining genomic risk scores, microbiome profiles, and real-time patient-reported outcomes.
• Cross-disciplinary consortia leveraging big-data analytics to refine evidence-based guidelines.The trends and hotspots identified in this analysis are consistent with broader movements in dental research towards precision medicine, technological integration, and holistic patient care. Previous studies have also highlighted the bidirectional relationship between orthodontics and periodontology, emphasizing the need for integrated treatment planning. However, this bibliometric approach provides a more nuanced and quantitative assessment of the field, offering insights that complement qualitative reviews and clinical studies. While this study provides valuable insights, it is subject to certain limitations. The reliance on Web of Science and PubMed databases may exclude relevant studies indexed elsewhere, potentially affecting the comprehensiveness of the analysis. Additionally, bibliometric indicators such as publication and citation counts do not fully capture the quality or clinical impact of individual studies. Future research could incorporate additional databases and qualitative assessments to provide a more exhaustive understanding of the field.

## Data Availability

The raw data supporting the conclusions of this article will be made available by the authors, without undue reservation.
